# Leads Veterinary Journalism in America

**Published:** 1898-03

**Authors:** 


### LEADS VETERINARY JOURNALISM IN AMERICA.

Our esteemed contemporary, the *Review*, seems much distressed over the cancellation of the Journal's headlines on its monthly wrappers. For the enlightenment of our editorial colleagues, we would say that we found ourselves confronted by the possibility of having our monthly edition excluded from the mails as third-class matter with anything but the name and address upon our wrappers. And, from the many inquiries in the past when the Journal was a day or two late in reaching ite readers, we did not desire to be overwhelmed with inquiries and complaints from them. The Journal still leads, and rejoices to see our contemporary following, in adding to its attractiveness in typographical character, better paper, our envelope-plan of wrapper, and its increased scope of news and information. The Journal has added one additional triumph in placing itself on a cash subscription basis. We no longer desire to take any advantage of our readers by continuing the Journal after the period paid for has elapsed, and then annoying our subscribers with dunning letters for their unpaid subscriptions, as well as unauthorized continuance of the same. With the same support and aid of our contributors, we shall endeavor to continue to advance and enhance veterinary journalism in America.

